# Estimation of the Proportion of Underachieving Students in Compulsory Secondary Education in Spain: An Application of the Rasch Model

**DOI:** 10.3389/fpsyg.2016.00303

**Published:** 2016-03-04

**Authors:** Alejandro Veas, Raquel Gilar, Pablo Miñano, Juan-Luis Castejón

**Affiliations:** Developmental Psychology and Didactics, University of AlicanteAlicante, Spain

**Keywords:** underachievement, high school, Rasch models, Differential Item Functioning, construct comparability approach

## Abstract

There are very few studies in Spain that treat underachievement rigorously, and those that do are typically related to gifted students. The present study examined the proportion of underachieving students using the Rasch measurement model. A sample of 643 first-year high school students (*mean age* = 12.09; *SD* = 0.47) from 8 schools in the province of Alicante (Spain) completed the Battery of Differential and General Skills (Badyg), and these students' General Points Average (GPAs) were recovered by teachers. Dichotomous and Partial credit Rasch models were performed. After adjusting the measurement instruments, the individual underachievement index provided a total sample of 181 underachieving students, or 28.14% of the total sample across the ability levels. This study confirms that the Rasch measurement model can accurately estimate the construct validity of both the intelligence test and the academic grades for the calculation of underachieving students. Furthermore, the present study constitutes a pioneer framework for the estimation of the prevalence of underachievement in Spain.

## Introduction

The concept of underachievement has been widely studied in the educational field in the last 50 years, showing a clear impact in high education studies and in professional careers (Conklin, 1940; Shaw and McCuen, 1960; Gurman, 1970; Delisle and Berger, 1990; Rimm, 1997; Smith, 2005; McCoach and Del Siegle, 2011). In the scientific literature, there is a general agreement that underachievement is the discrepancy between what can be expected and what is actually achieved (Phillipson, 2008). However, it is important to note that there is not a unique and accepted definition, due to conceptual problems mainly related to the arbitrary operationalization of the discrepancy between ability and achievement (Ziegler et al., 2012). This fact has resulted in a diversification of studies that can include from students with emotional and behavior disorders (Lane et al., 2002) to students with learning disabilities (Fletcher et al., 2005).

Including or not these kind of diversifications, the consequences of being underachieving could imply insufficient support (Ziegler et al., 2012), low academic self-perceptions (Matthews and McBee, 2007) or low goal-valuation (McCoach and Siegle, 2003; Baslanti and McCoach, 2006), among other negative processes (McCall et al., 2000).

In Spain, the percentage of school failure or dropout (those students who leave the educational system) during the course of 2012–2013 was 23.5% (Eurostat, 2014), which is double of the percentage in the European Union, 11.9% for the same period. Some communities in Spain even reached 29.8 %. This considerable percentage of students that leads to school failure could be related to underachievement.

The estimation of the percentage of underachieving students can vary, depending on some aspects such as the operational underachievement definition or the socio-cultural context of students involved. For example, Rimm (1987) made an estimation of 50% of students with low achievement and high potential in Elementary Secondary Education, whereas Colangelo et al. (2004) made a lower estimate of 10% in a sample of high school students. In China, Phillipson (2008) calculated an empirical percentage of underachieving students that moved from 10% in the 50–59 capacity percentile bands (measured by a frequency distribution of the difference between ability and potential), to 32% in the higher 95 percentile bands in Primary Education. In Secondary Education, the percentage of underachieving students reached 53% in those whose capacity was in the higher bands. This implies that, with increasing capacity, the percentage of underachieving students is considerably higher, and this is more visible in Secondary Education.

There are hardly any studies in Spain that treat underachievement rigorously, and they are usually related to gifted students. One of the most important studies was developed in Madrid by García-Alcañiz (1991), where the percentage of gifted students with school failure was 30%, similar to normal population. Jiménez and Álvarez's (1997) confirmed the same percentage of students with high IQ and low achievement during the first school years. Broc (2010) treated underachievement in the context of school failure and absenteeism, and formulated a theoretical model regarding the reasons for low academic achievement in students with high potential levels.

With respect to the operational definition of underachievement, the discrepancy between the potential ability and the academic achievement is, in some cases, restricted to gifted students, as happens frequently in the United States (Reis and McCoach, 2000; Siegle et al., 2006), whereas the studies from China are opened to all of the ability ranges (Phillipson, 2008, 2010). The second perspective could suppose a more adequate and individualized response to all students.

From a methodological perspective, some questions have been raised about the adequacy of different identification methods proposed in the studies. Traditionally, there have been three statistical methods: the absolute split method, the simple difference method and the regression method (Plewis, 1991; Lau and Chan, 2001). According to Phillipson (2008), *leaving aside arguments against the arbitrary use of cut-scores such as top 25% and standard deviations of 1, all three methods are highly dependent on sample parameters such as the means and standard deviations*. This assertion implies that the use of statistical parameters such as the means and standard deviations would be inaccurate whether we want to evaluate individual comparisons.

The more recent method is based on the application of the Rasch model (Phillipson and Tse, 2007; Phillipson, 2008). This method is most well-known among item response theories (Rasch, 1960/1980; Wright and Stone, 1979), representing the variability of a construct based on the calibration of ordinal data from a shared measurement scale. The Rasch model establishes that the difficulty of the items and the ability of the subjects can be measured on the same scale and that the likelihood that a subject responds correctly to an item is based on the difference between the ability of the subject and the difficulty of the item. Both measures (ability and difficulty) are estimated using logit units because the scale used by the model is logarithmic. Using the same measurement scale establishes homogenous intervals, which means that the same difference between the difficulty parameter of an item and the ability of a subject involves the same probability of success along the entire scale (Preece, 2010).

While many statistical models try to fit the model to the data, the opposite occurs in the Rasch measurement model. That is, the data must fit the model to be accepted (Bond and Fox, 2001), as the model provides detailed information about the interaction between persons and items. This adjustment can be conducted using residual measures, i.e., the difference between a subject's response to a given item and the expected response calculated by the model. The adjustment measures can be standardized for a particular item or subject in two ways (Bond and Fox, 2007). On one side is Outfit, which is the root mean square of the residuals, divided by the degrees of freedom. This measure can be interpreted as an overall measure that expresses whether the answers given to a particular item will fit the model. On the other side is Infit, a measure that eliminates the extreme scores that influence the Outfit by using the residuals of individuals whose ability levels are in the closest range to a particular item.

Statistical Infit and Outfit are calculated based on root mean squares, depending on the statistical value of Pearson's chi-squared divided by the degrees of freedom, thus forming a scale with values that can range from 0 to infinity. Values below 1 indicate a higher than expected fit of the model, while values greater than 1 indicate a poor fit of the model. If we have an Infit value of 1.40, then we can assert that there is 40% more data variability compared to the model's prediction. An Outfit of 0.80 indicates that 20% less data variability is observed with respect to the model's prediction.

Phillipson (2008) performed the calculation of Chinese underachieving in mathematics by scaling the responses to both the Raven Progressive Matrices (RPM) and the Hong Kong Attainment Test (HKAT). The main purpose of this study is to develop a psychometric model for the detection of underachievement, based on the use of academic grades under the construct comparability approach (Coe, 2008). In this sense, we propose a calculation of general underachievement by scaling the responses to both the Battery of Differential and General Skills (Badyg) and General Points Average (GPAs).

In Spain, the educational evaluation processes undertaken by teachers in schools are based on conducting non-standardized written tests and the assessment of attitudinal variables, (e. g., quality of the participation in the proposed activities) observed in the classroom. Thus, the application of the evaluation criteria leads to a total grade for each of the courses which the student is enrolled. Therefore, the use of academic grades are quite important, as schools continue to evaluate skills through other traditional methods and/or measurement instruments, such as written exams, oral exams, group work, etc., that are based on the evaluation criteria of regional regulations.

On the other hand, there are a significant number of studies on academic performance that have used the results of studies at the international level, such as the Trends in International Mathematics and Science Study (TIMSS) and especially the Program for International Student Assessment (PISA) by using standardized tests (Ruiz de Miguel, 2009; Calero et al., 2010; Ferrera et al., 2012). However, these tests do not evaluate curricular content but instead mastery and the understanding of problems and concepts, in addition to the ability to adapt to different situation. As such they are conceptually different from the evaluative approach in use in schools (Anagnostopoulou et al., 2013; Cordero et al., 2013). At this point, although academic grades and test performance have to be seen as complementary (Marrero and Espino, 1988), it is possible (especially in Spain) that academic grades emerge as the most valid values of a student's current level of achievement because they evaluate academic contents within a classroom environment (Marzano, 2000; McCoach and Del Siegle, 2011).

The analyses of the conceptual and methodological processes in comparing school grades have been studied extensively in the last quarter of the twentieth century, especially in the United Kingdom (Forrest and Vickerman, 1982; Fitz-Gibbon et al., 1994; Goldstein and Cresswell, 1996; Goldstein and Thomas, 1996). Furthermore, in most recent years, a new conceptualization of the term *comparison* has emerged, named the construct comparability approach (Coe, 2008). This model indicates that when comparing any two elements, they must have something in common to serve as the basis for this comparison. Just as two tests can be compared based on their measurements using the same scale (Muñiz, 1997), in the context of comparing academic grades, we can only compare those that measure a shared construct, which in our case is academic performance. Therefore, the premise of this approach would be as follows (Coe, 2008): Two grades from two students are comparable if the performance of both students, which corresponds to the same level of the latent construct that they share, leads to the same grade.

According to this postulate, the difficulty of a course will correspond to a specific level established in the latent variable. A course will be more difficult than another to the extent that a higher level of performance or ability is needed to achieve the same grade. If the latent construct is changed, this relationship may easily be the inverse (Coe, 2010).

The measurement of comparability would be based on using the grades from the courses as a measurement to validate the construct, which implies that they must provide good levels of content representativeness, good internal consistency, and appropriate levels of correlation between the variables that comprise the different courses. If, in studies on academic performance or other research topics, the mean grades of the courses are used to obtain the academic performance variable, then it is essential to use statistical tools to confirm their fit from the measurement standpoint.

As noted above, at this level of analysis we start with considering each of the courses as a test with specific items, with the range of grades from 1 to 10, which implies various degrees or categories of success. The partial credit model (Wright and Masters, 1982) enables an analysis of the difficulty of achieving a specific score for each of the courses separately, following the Rasch methodology with polytomous data. Moreover, Rasch models for dichotomous data, such as the Badyg, are based on items that are scored as either correct or incorrect.

At this point, it becomes necessary to test the extent to which students can be identified as underachieving and non-underachieving by using measures in the same metric scale. Therefore, the present study will describe an estimation of the proportion of underachieving Spanish students in the first course of compulsory secondary education. Rasch measurement method will ensure an estimation of the construct validity of both the intelligence test and the academic grades.

## Methods

### Participants

Random cluster sampling was used, using the school as the sampling unit, taking into account geographical areas of the province of Alicante. A total of 8 schools in the province of Alicante were included; 2 schools were private, while the rest were public. A total of 643 students in the first year of Compulsory Secondary Education (Educación Secundaria Obligatoria—E.S.O.) participated in the study. Twenty nine students (4.31%) were excluded from the final sample due to having an insufficient command of the language, because they had special educational needs, or because they did not have parental consent. Fifty one percent of the students were male, and 49% were female, with an average age of 12.09 years and a standard deviation of 0.47. Five hundred twenty three participants (81.4%) were enrolled in a public school, while 120 (18.6%) were enrolled in a private school. Overall student in each class in each school took part in the study. Because of the racial and ethnic homogeneity of the country, the majority of children were Caucasian (98%). Childhood socioeconomic status (SES) was indexed according to parental occupation. There was a wide range of socioeconomic status with a predominance of middle class children. This classification was based on the level of incomes and the level of studies of the families. The regional education counselors determined SES through a questionnaire registered with the responses of the students. The variable used were: parents' professions, professional situation and level of studies, number of books at home, cultural and sporting activities, and availability of technological means at home.

Chi-square test was used to determine whether there were differences between the gender of the sample (51.2% boys and 48.8% girls) and the gender of the national student population (51.3% boys and 48.7% girls), supporting the absence of gender differences between sample and population (χ^2^ = 0.29, *df* = 1, *p*>0.05).

In the sample, the percentage of students who assist to public schools (81.4%) was slightly higher than the percentage who assist to private schools (18.6%) in the population, which was 76 and 24% respectively (χ^2^ = 4.1, *df* = 1, *p*>0.01); although there were no differences in the private/public school ratio (χ^2^ = 2.67, *df* = 1, *p*>0.05).

So, in general terms, the sample studies was representative of the national general population of first grade Compulsory Secondary Education students.

### Measures

For the analysis of academic performance, numerical GPAs from 9 mandatory courses, which the faculty provided at the end of the school year, were considered. The courses recorded were Spanish Language and Literature, Natural Sciences, Valencian Language, Social Sciences, Mathematics, English, Technology, Art Education, and Physical Education. Student scores showed high reliability, with a Cronbach's alpha of 0.93. Students' scholar ability was estimated using the Battery of Differential and General Skills (Yuste et al., 2005) or Badyg. This Spanish battery measures the capacities and academic abilities of students. There are six subscales: Analogies (A), Series (S), Matrices (M), Complete (C), Problems (P), and Figures fit (E). Each subscale is measured with 32 items with five response options and only one option is correct, producing a total of 192 items. For this study, Cronbach's alpha values for each subscale were 0.83, 0.89, 0.79, 0.83, 0.77, and 0.87 respectively. Furthermore, a general intelligence quotient (IQ) could be obtained based on the punctuations from the distinct differential skills. The Cronbach's alpha of the total IQ was 0.83.

### Procedure

Prior to data collection, the necessary permission was requested from the educational administration and school boards of the various schools. After obtaining these permissions, the parents or legal guardians of the students had to provide the corresponding informed consent. Data collection was performed in the schools themselves during the second trimester of the school year and during normal school hours. The data were collected by collaborating researchers previously trained in the standards and guidelines for data collection.

### Data analysis

For this study, punctuations from Badyg and school grades were analyzed using Winsteps version 3.81 statistical software (Linacre, 2011), whose estimates were based on the joint maximum likelihood (Bond, 2003; Linacre, 2012).

From the maximum likelihood procedure, it is possible to obtain a value for the difficulty of a certain item that best explains the pattern of recorded performance. Similarly, one can obtain a value for the ability of each individual depending on the pattern of the indices of difficulty. This process is repeated continuously using the most recent estimates of skill and difficulty until the estimate converges.

Once fit indices from both measures have been observed, the Rasch model allows for the testing of the hypothesis that two tests measure the same underlying construct (Bond and Fox, 2001, 2007). This comparison is tested by elaborating a scatter plot of students' Rasch responses to both tests and to observe whether the points lie between 95% confidence bands (Phillipson, 2008). Those points outside the 95% confidence bands indicate that the achievement level is not what is expected.

## Results

Taking into account that school grades do not constitute a validated test, a deeper analysis of the fit of the courses has been conducted, based on the inter-subject comparability approach (Tasmanian Qualification Authority, 2006, 2007; Coe, 2008; Korobko et al., 2008). Table 1 shows the courses analyzed, the indices of fit, and the item-scale correlation. The statistics for fit are very important when deciding whether the items follow the proposed Rasch model. However, the interpretation of these cases is often complex due to the absence of unanimity in setting minimum thresholds (Smith et al., 1998). In our case, we used an approximate range of 0.8–1.2 for Infit and Outfit (Bond and Fox, 2007, pp. 243), in addition to the observation of each of the item characteristic curves (ICCs). Table 1 shows a lack of fit in a number of courses (Spanish Language and Literature, Natural Sciences, Valencian Language, and Physical Education), which assumes a lack of fit for the subjects' pattern of responses with respect to the model. Furthermore, in the ICCs of most of these items, the highest response probabilities are exceeded by adjacent categories, especially the lowest categories. The latter also have a fairly low number of subjects. Therefore, this situation implies that the pattern of responses does not adequately fit the model and that the reconversion of the performance categories for all courses may be appropriate.

**Table 1:** **Statistics of fit for the courses in the first and second grades in ESO**.

**Courses**	**Count**	**Infit**	**Outfit**	**Item-scale correlation**
Spanish Language and Literature	643	0.63	0.63	0.88
Natural Sciences	642	0.62	0.62	0.87
Valencian Language	625	0.71	0.71	0.86
Social Sciences	640	0.88	0.86	0.85
Mathematics	641	0.94	0.93	0.85
English	629	1.16	1.13	0.82
Technology	640	1.12	1.11	0.78
Arts Education	642	1.20	1.21	0.77
Physical Education	641	1.53	1.87	0.64

Based on the qualitative scores of Spanish schools, recoding was performed using the following values: 1 for categories 1, 2, 3, and 4 (“poor”); 2 for categories 5 and 6 (“sufficient” and “good”); 3 for categories 7 and 8 (“notable”); and 4 for categories 9 and 10 (“outstanding”).

The new calibration of the courses provided a good fit for the data (Table 2), except for physical education (Infit = 1.43; Outfit = 1.52). The analysis of Differential Item Functioning (DIF) estimated the distribution of the difficulty parameter in the sample of boys and girls. The results show that the course Visual Arts Education is easier for girls and that the difference is statistically significant (*Mantel* χ^2^ = 23.518; *p* = 0.000). The differences found in Valencian Language, mathematics, social sciences, and natural sciences were not statistically significant, with *p* > 0.001. Therefore, both Physical Education and Visual Arts Education were eliminated to estimate the new model.

**Table 2:** **Indices of fit for first year of ESO with recoded values**.

**Courses**	**Count**	**Infit**	**Outfit**
Spanish Language and Literature	643	0.75	0.79
Natural Sciences	642	0.75	0.75
Valencian Language	625	0.78	0.76
Social Sciences	640	0.83	0.83
Mathematics	641	0.94	0.99
English	629	1.03	1.04
Technology	640	1.13	1.12

For the analysis of unidimensionality, a principal component analysis of the residual scores was conducted (Linacre, 1998). The results showed a principal factor that was able to explain 69.3% of the variance of the latent trait, with a wide difference between the weight of the first factor and the next (*Eigenvalue* = 1.4), which favors the unidimensionality of the model.

Although not shown, each of the Badyg blocks was analyzed separately. The item analyses for the Badyg demonstrate that all items except for items 1M, 11M, 7M, 2E, 13E, 29E, 2P, 8P, and 29S have an Infit Mean SQ between 0.80 and 1.20, indicating that the majority of items fitted the model satisfactorily. As regards person fit, the majority of Infit and Outfit Mean SQ values of persons are within values of 1.3. Approximately 95% of students fit the Rasch model (Bond and Fox, 2001, pp. 176–177; Phillipson and Tse, 2007).

Table 3 shows the summary of the item and person estimates for the Badyg and school grades. For the Badyg, the mean logit of the items is 0.00, and the majority of the items are within a SD of 1.22. The reliability of the estimate, similar to Cronbach's alpha, is 0.99, which indicates that Badyg is a useful test. Infit and Outfit Mean have values close to 1, demonstrating that the data fits the Rasch model very well. The mean person ability estimate has a value of −4.44 (SD = 0.75), meaning that these students find the Badyg more difficult, as expected. The reliability of the estimate has a strong value of 0.95, and the values of Infit and Outfit Mean are close to 1.

**Table 3:** **Summary of item and person estimates from Badyg and school grades**.

	**Badyg**		**School grades**	
	**Item**	**Person**	**Item**	**Person**
**LOGIT SUMMARY**				
Mean	0.00	−4.44	0.00	1.05
SD	1.22	0.79	0.27	1.33
Reliability of estimate	0.99	0.95	0.99	0.92
**FIT STATISTICS**				
Infit Mean SQ				
Mean	1.01	1.01	0.98	0.98
SD	0.13	0.31	0.28	0.66
Outfit Mean SQ				
Mean	1.04	1.04	1.01	1.01
SD	0.18	0.41	0.37	0.72

For the School grades, the mean (and SD) logit for items is 0.00 (0.27), showing that grades are not widespread in the interval scale. The reliability of the estimate is very high, with a value of 0.99. Infit and Outfit Mean (and SD) have values close to 1, which implies a good fit of the data. The mean (and SD) of the person estimate from the school grades are (1.05 and 1.33). In this case, students find the majority of the courses easy.

**Figure 1:**
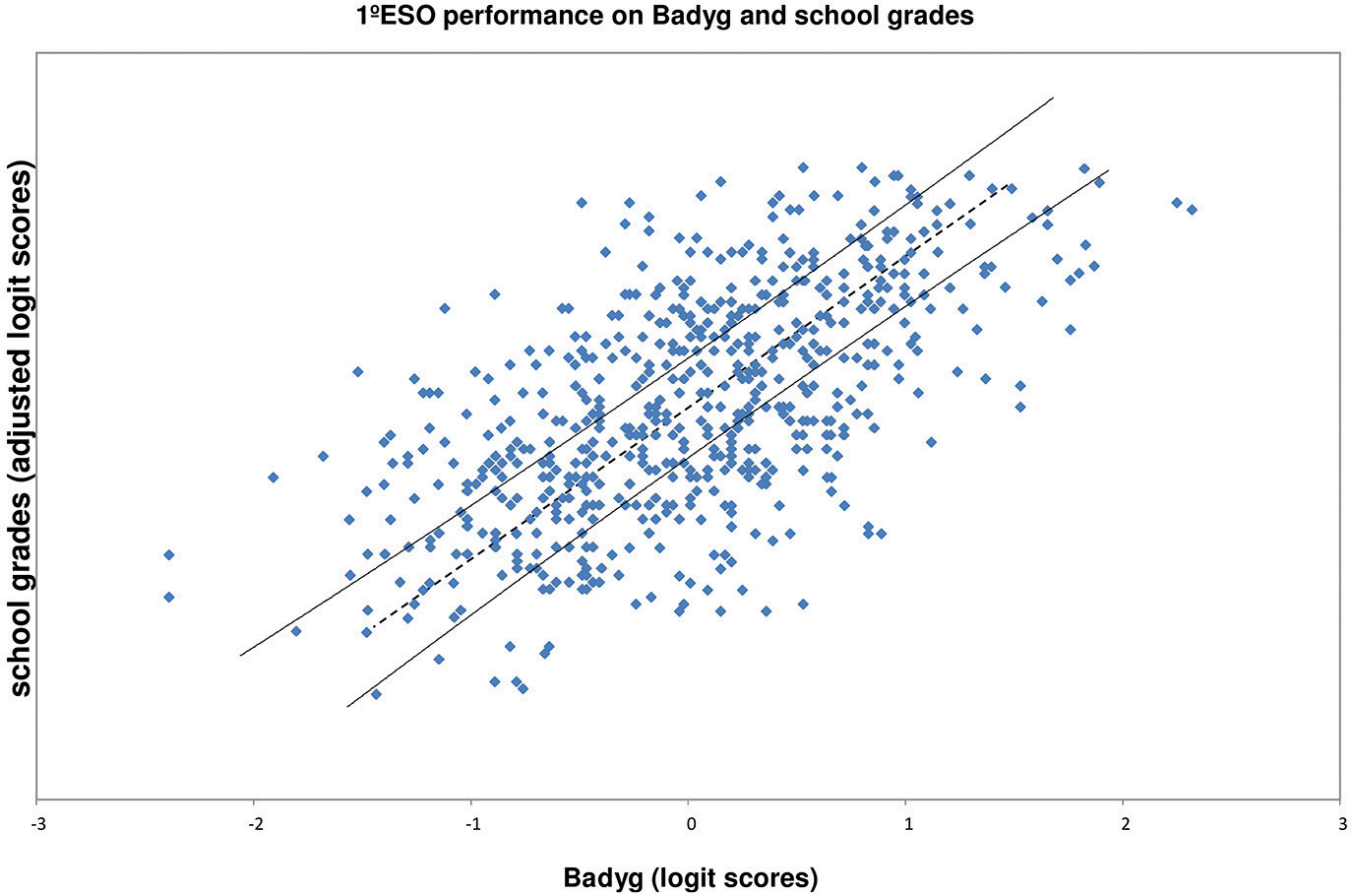
**Person logit school grades scores and person logit Badyg scores, with 9.5% confidence bands**.

After adjusting the school grade scores and Badyg scores to be aligned with mean 0 and SD 1, the scatterplot of person logit school grades scores and person logit Badyg scores was produced (Figure 1), using the 95% confidence bands (Bond and Fox, 2001, p. 57). Points within the bands include students with normal achievement. Points below the lower band represent students whose school grades are significantly lower than expected by their Badyg score. Finally, points above the upper band represent students whose school grades are significantly higher than expected by their Badyg score.

The individual underachievement index, based on the significant differences between GPA and Badyg, provides the exact number of underachieving students, 181 or 28.14% of the total sample of 643 students across the ability levels. From the total of underachieving students, 29 were enrolled in private school (16.02%), whereas 152 students were enrolled in public school (83.98%). The analysis of the differences between these percentages of underachieving students identified in public and private schools showed that these differences were statistically significant (χ^2^ = 17.13, *df* = 1, *p* = 0.00). With respect to gender differences between underachieving and non-underachieving students, these was also statistically significant (χ^2^ = 6.24, *df* = 1, *p* = 0.012). A higher percentage of boys (33.4%) was detected as underachieving in comparison with girls (24.4%), whereas the opposite occurs in the non-underachievement group (66.6% boys and 75.6 girls).

## Discussion

The present study describes an estimation of the proportion of underachieving Spanish students in the first course of compulsory secondary education. In light of the results, we may assert that the proportion of underachieving students found in the sample with the Rasch method is relatively high, with a value of 181, or 28.14% of the total sample. Moreover, important gender differences are observed between non-underachieving and underachieving students with the total sample. A higher proportion of boys are identified as underachieving in comparison with girls. These results with the Rasch method is consistent with previous results using other methods of measuring underachievement (Gibbs et al., 2008).

This percentage is similar to those found previously in Spain. Jiménez and Álvarez's (1997) confirmed the presence of students with high IQ and low achievement since the first school years, showing a percentage of 30%. It seems that the percentage of Spanish underachieving students is relatively higher than in other countries. Colangelo et al. (2004) considered that the percentage of underachieving students in the United States is near 10%. In China, Phillipson (2008) found close to 12% of underachieving students in a large sample in the normal capacity band. The use of the Rasch method in our study estimates more students as underachieving's, in comparison with the traditional methods employed by Lau and Chan (2001). This fact could be related to that this model does not establish an arbitrary cut-off for the selection, and use a logarithmic scale in where both measures are fitted and adjusted.

With respect to the high number of underachieving students, it is important to consider the contextual factors in the present study. Firstly, it seems that underachievement changes from more general to more subject-specific areas at the end of elementary school (McCall et al., 2000). Secondly, some evidence from the United States highlights the importance of the change to secondary education (Eccles and Roeser, 2011). In this sense, it is possible that this level of underachieving is affected by this transition of Primary to Secondary Education, given that the sample employed corresponds to the first year of the Compulsory Secondary Education. The start of the Secondary Education constitutes a new educational stage in Spain with some important changes, such as the change of school. This implies that this transition is normally related to a difficult process in our educational context (Pérez and Castejón, 2008) as happens in other educational systems (Eccles and Roeser, 2011). It is possible that underachievement declines as students adjust to this transition, which can be analyzed in future studies by including students from higher levels of Secondary Education.

Some points must be addressed in the present study, as they can affect the levels of detection of underachievement. First, we referred to a global underachievement instead of an underachievement index in a specific area, which implies a major probability of obtaining a higher number of underachieving students. Second, and according to previous studies (Phillipson and Tse, 2007), the number of underachieving students can vary depending on the method employed. In the present study, the Rasch method is used as enhanced objective and non-sample dependent measures when comparing the degree of agreement between two tests (Bond and Fox, 2007). Therefore, it is possible to know the lack of concordance between these two tests (Phillipson, 2008), in this case Badyg and GPA, when exploring the underachievement patterns at the individual level.

The analysis of GPA through the partial credit model confirmed the possibility of comparison, based on the construct comparability approach (Newton, 2005; Coe, 2008). It was necessary to reduce the number of categories for all courses and eliminate the Physical Education course to obtain adequate levels of fit (Wright and Masters, 1981; Wright, 1984; Wright et al., 1994) and the Arts and Visual Education course because it had a significant DIF. The courses analyzed together aim at measuring overall academic performance, showing optimal values of factor loadings in the principal component analysis, and confirming the unidimensionality of the construct. As shown, the partial credit model performs a calculation of the difficulty indices for each course that allow us to know the ability level required by the subject to achieve a certain grade. This model has been widely used in education because it is a highly effective analysis tool (Bond, 2003).

For a more objective measure of the courses, it would be advisable to reduce the number of grades for evaluation, especially in the lowest categories. In the present study, we found that in all high schools analyzed, the grades 1, 2, and 3 are assigned to a very low proportion in all courses. In addition, a wider range of grades leads to a more heterogeneous distribution of evaluation criteria than the standards indicate. In this regard, schools in countries such as the United Kingdom use small grade ranges (Department for Education, 2013).

In addition, some limitations may need to be addressed in the future. Firstly, existence of cultural factors must be added in future studies (Reis and McCoach, 2000). Statistical differences have been detected in the number of underachieving students attending private school with those who attend the public school. Therefore, it is necessary to develop studies in Spain that are focused on estimating the percentages of underachieving students with a larger sample and in our socio-cultural context. Furthermore, it would be interesting to compare different identification methods in order to obtain more reliable percentages of underachieving students. Secondly, it would be necessary to employ achievement tests in future studies in order to contrast the quality of the use of grades in Spanish schools when detecting underachieving, and more specifically, in public and private schools.

Another important point is that this study focuses on students of all intelligence levels, not only on gifted students. Therefore, the heterogeneity level could be higher (Reis and McCoach, 2000; Siegle and McCoach, 2005; Phillipson and Tse, 2007). Different aspects such as educational level, gender, and other individual aspects can introduce characteristic patterns associated with different subpopulations. In future studies, it is necessary to employ methodological processes that can detect this heterogeneity (Madigson and Vermunt, 2002; Lubke and Munthén, 2005) and to establish whether underachievings' subgroups exist across the ability levels. In this line, Reis and McCoach (2000) describe six different types of underachievings: Anxious Underachievings, Wheeler-dealer Underachievings, Coasting Underachievings, Defiant Underachievings, Identity Search Underachievings, and Sad or Depressed Underachievings. In more recent studies, Snyder and Linnenbrink-García (2013) propose that there are multiple developmental trajectories in underachievement, and Ritchotte et al. (2014) find two types of underachieving students: students having less positive attitudes toward their self-efficacy, the meaningfulness of tasks, their school environment and their self-regulation skills, and on the other hand students having more positive attitudes toward these constructs.

The present study constitutes a pioneer analysis for the estimation of the prevalence of underachievement in Spain. These results could be useful to educational orientation and the instructional interventions performed by teachers, as they have already done in other countries (McCall et al., 2000). In these situations, once the Rasch method provides individual detections of underachievement, the education professionals could help the student value his/her academic goals and learning strategies (Chan, 1999; McCoach and Siegle, 2003; Obergriesser and Stoeger, 2015), get self-regulated strategies (Reis and Greene, 2014), develop positive attitudes toward school and teachers, and adequate and enhance self-concept. Furthermore, to obtain a deeper educational treatment, it is necessary to introduce differential variables among groups (underachieving, normal-achieving and overachieving), such as learning strategies, self-concept, parent involvement and social acceptance among peers, apart from motivational and attitudinal variables. Baker et al. (1998) suggest that, especially in adolescents, the confluence of all of these variables is what explained most of the underachievement.

## Author contributions

AV Theoretical review of the topic. Rasch Analysis of the measures. Differential item functioning of each test. RG Theoretical review of the topic. Review of the references. PM Theoretical review of the topic. JC Quantitative methods. Analysis of the sample. Reliability of the instruments.

## Funding

The present work was supported by the Spanish Ministry of Economy and Competitiveness (Award number: EDU2012-32156) and the Vice Chancellor for Research of the University of Alicante (Award number: GRE11-15). The corresponding author is funded by the Spanish Ministry of Economy and Competitiveness (Reference of the grant: BES-2013-064331).

### Conflict of interest statement

The authors declare that the research was conducted in the absence of any commercial or financial relationships that could be construed as a potential conflict of interest.
